# Pancreatic Cancer-Associated Pain Management Beyond Opioids: Integrating Oncological Treatment, Palliative Care, and Interventional Pain Medicine

**DOI:** 10.3390/cancers18193127

**Published:** 2026-09-27

**Authors:** Ferenc Zelenai, Eszter Bakos, Villő Süller-Torma, Magdolna Dank, Ildikó Madurka, Orsolya Horváth

**Affiliations:** 1 Department of Anesthesiology and Intensive Care, Pain Unit, National Institute of Oncology, 1122 Budapest, Hungary; madurka.ildiko@oncol.hu; 2 Palliative Care Unit, National Institute of Oncology, 1122 Budapest, Hungary; suller.torma.villo@oncol.hu (V.S.-T.); horvath.orsolya@oncol.hu (O.H.); 3 National Institute of Oncology, 1122 Budapest, Hungary; dank.magdolna@oncol.hu

**Keywords:** pancreatic cancer, cancer pain, oncological treatment, palliative care, pharmacological pain management, interventional pain management, multidisciplinary care, integrated care

## Abstract

Pain is one of the most frequent and burdensome symptoms of pancreatic cancer and may substantially impair quality of life throughout the disease trajectory. Its complex and evolving etiology, involving visceral, neuropathic, somatic, and treatment-related mechanisms, frequently makes adequate pain control challenging. This critical narrative review provides a multidisciplinary overview of pancreatic cancer-associated pain management, integrating oncological treatment, palliative and pharmacological care, and interventional pain management into a practical clinical framework. Anticancer therapies, including chemotherapy and radiotherapy, may provide clinically meaningful analgesia in addition to their disease-controlling effect. Early integration of palliative care enables repeated symptom assessment, individualized pharmacological treatment, and management of the psychological, social, and spiritual dimensions of total pain. When conventional analgesic strategies provide insufficient relief or cause intolerable adverse effects, interventional techniques such as coeliac plexus neurolysis, splanchnic neurolysis, or intrathecal drug delivery can complement ongoing oncological and palliative treatment. Rather than following a rigid stepwise analgesic approach, pancreatic cancer-associated pain management should be individualized according to pain mechanism, disease stage and anatomy, previous treatments, functional status, expected survival, and patient preferences. Based on the available evidence and multidisciplinary clinical considerations, we propose a practical patient-centered pathway that dynamically integrates disease-directed, pharmacological, supportive, and interventional strategies throughout the disease course. Early multidisciplinary collaboration and continuous reassessment may facilitate timely treatment escalation and improve pain control while minimizing treatment burden.

## Simple Summary

Pain is one of the most common and burdensome symptoms of pancreatic cancer and can substantially reduce patients’ quality of life. Its management is challenging because pain may result from the tumor itself, cancer treatment, or disease progression and may change throughout the course of the disease. This review aims to provide a practical, multidisciplinary overview of pancreatic cancer-associated pain management by integrating three complementary perspectives: oncological treatment, palliative and pharmacological care, and interventional pain management. Available evidence indicates that anticancer therapies can contribute to pain relief, while early palliative care and individualized pharmacological treatment remain essential throughout the disease course. Interventional procedures may provide additional options when pain remains inadequately controlled or treatment-related adverse effects become limiting. We propose a practical patient-centered approach, in which these strategies are not regarded as isolated or strictly sequential treatments but are dynamically combined according to disease stage, pain characteristics, previous treatments, and individual patient needs.

## 1. Introduction

Pancreatic cancer is one of the most lethal malignancies and represents a growing health burden worldwide. Although it accounts for a relatively modest proportion of all newly diagnosed cancers, its mortality remains disproportionately high, largely because of late diagnosis, aggressive tumor biology, and the limited proportion of patients eligible for potentially curative surgical resection [1]. Recent estimates from the European Cancer Information System (ECIS) indicate that pancreatic cancer accounts for approximately 8% of all cancer deaths in the European Union, placing it among the four leading causes of cancer mortality [2]. Despite advances in systemic oncological therapy, surgery, radiotherapy, and supportive care, pancreatic cancer therefore remains a malignancy in which symptom control and preservation of quality of life are major components of patient management.

More than 60% of pancreatic cancer patients experience pain at the time of the diagnosis, while 70–80% of patients report pain during the disease trajectory [3,4,5]. The pathophysiology of pain in pancreatic cancer is complex. Visceral nociceptive signals are transmitted by the sympathetic nervous system through the splanchnic nerves and the coeliac plexus. Tumor expansion causes tissue injury, ductal obstruction, and can infiltrate or compress adherent structures. Perineural invasion is a characteristic feature of pancreatic ductal adenocarcinoma and is associated with both disease progression and pain generation. Neurotrophic factors further contribute to tumor–nerve interactions and nociceptive sensitization. In particular, nerve growth factor (NGF), acting predominantly through tropomyosin receptor kinase A (TrkA) and p75 neurotrophin receptor (p75NTR), promotes neural remodeling and perineural invasion and can increase the sensitivity of nociceptive afferents. Increased NGF/TrkA signaling has also been associated with greater pain severity in pancreatic cancer, providing a molecular link between tumor progression, neural alterations, and pain generation [6]. Consequently, pain in advanced pancreatic cancer frequently has mixed visceral, somatic, and neuropathic components rather than representing a purely visceral pain syndrome [3]. Pain can substantially impair sleep, mobility, appetite, and physical functioning; hence, pain control has to be an integrative part of the care.

Pharmacological management remains fundamental in cancer related pain; however, the need for repeated opioid escalation due to disease progression contributes to constipation, nausea, sedation, and cognitive impairment [7]. Interventional pain management procedures are able to relieve pain without the side effects of opioid therapy; however, they carry procedural risks [8]. Integration of palliative care is an essential component of multimodal pain management in pancreatic cancer, as it facilitates systematic symptom assessment, optimization of analgesic therapy, and management of the physical and psychosocial factors contributing to pain [9].

The aim of this critical narrative review is to provide an integrated, multidisciplinary overview of contemporary pain management in adult patients with pancreatic cancer. We examine available therapeutic strategies from the complementary perspectives of oncology, palliative medicine, and interventional pain management. Less widely recognized topics that we consider particularly important, such as psycho-social care and interventional pain management, are discussed in greater detail, whereas more established areas, such as pharmacological therapy, are addressed more concisely.

## 2. Methods

A comprehensive search of the PubMed database was conducted from January 2016 to July 2026. Search terms were selected in the context of pancreatic carcinoma according to the relevant fields of interest, including palliative care (“palliative care”, “early palliative care”, “palliative sedation”), oncology (“chemotherapy”, “radiotherapy”, “pain as clinical endpoint”), and interventional pain management (“coeliac plexus block”, “splanchnic nerve block”, “intrathecal”, “neurolysis”). Records were exported to Zotero, and in the merged libraries, duplicates were removed. Three authors independently screened the titles and abstracts against predefined eligibility criteria. Full texts of potentially eligible records were retrieved and assessed independently by the three authors. Eligibility criteria: Papers that specifically addressed the pancreatic cancer-associated pain or its management were included to ensure clinical relevance. In addition to database searching, we screened reference lists of included articles and relevant references were added as additional eligible studies (snowballing). As a critical narrative review the quality of included studies were assessed independently by the three authors. Although the primary search window was January 2016 to July 2026, we included a limited number of earlier publications identified through reference-list snowballing when they were assessed seminal or directly relevant to pancreatic cancer-associated pain management and not superseded by more recent evidence. These pre-2016 studies were included to provide essential background on established interventions.

## 3. Oncological Treatment as a Component of Pain Management

### 3.1. Chemotherapy

Evaluations of chemotherapy efficacy typically employ overall survival (OS) or progression-free survival (PFS) as primary endpoints, whereas pain and other clinical symptoms are generally evaluated as secondary endpoints. However, research indicates that patients with non-small cell lung cancer (NSCLC) generally favor therapies that improve overall quality of life -even without life extension- over treatments that offer only marginal OS benefits without symptomatic relief [10].

In a study analyzing 103 patients with advanced pancreatic cancer (APC), Kim et al. found that 17% of patients failed to achieve a radiological response (complete response [CR] or partial response [PR]) following chemotherapy, but experienced a self-reported improvement in pain and a reduction in analgesic opioid usage of at least 50% compared with baseline. The authors observed that patients who experienced pain reduction also achieved longer OS. Furthermore, clinical benefit—defined as pain reduction, an improvement in ECOG performance status (PS) of >7% weight gain—was significantly associated with a decreased risk of death, comparable to the prognostic impact of having locally advanced versus metastatic disease, or an ECOG PS of 0–1. These findings suggest that chemotherapy plays a crucial role not just in survival prolongation, but in alleviating pain caused by APC [11].

Another study evaluated the impact of nab-paclitaxel on quality of life (QoL). The authors investigated whether the reduction in cancer-related pain had a greater positive effect on patient QoL than the pain caused by chemotherapy-induced peripheral neuropathy. They found that, among patients with APC treated with the nab-paclitaxel plus gemcitabine protocol, 48% were able to reduce their use of analgesics, and 53.8% reported QoL improvements associated with pain alleviation. Additionally, 34% reported lower pain scores on the Visual Analog Scale (VAS) as a result of treatment [12].

In another patient cohort comprising individuals with potentially resectable pancreatic cancer, the effect of neoadjuvant FOLFIRINOX on QoL was investigated. Using pain-specific questionnaires, the researchers demonstrated a significant analgesic effect after 8 and 12 cycles of treatment [13]. Similar findings were reported by van der Sijde et al., who compared the QoL of patients with locally advanced pancreatic cancer (LAPC) receiving FOLFIRINOX to reference values from other patients with advanced cancer. They observed that although LAPC frequently causes severe pain, patients treated with FOLFIRINOX reported significantly lower pain levels compared to general cancer patients, patients with stage III–IV disease, and those with hepato-pancreato-biliary malignancies (reference values were obtained from the online dataset of the EORTC Quality of Life Group) [14].

In the NAPOLI-1 trial, fluorouracil + leucovorin + nanoliposomal irinotecan demonstrated OS and PFS benefits in patients pretreated with gemcitabine-based chemotherapy compared with fluorouracil + leucovorin [15]. In an analysis of the study’s QoL data, although pain relief was not addressed in detail, assessments using the EORTC QLQ-C30 questionnaire, which includes a pain subscale, indicated that the addition of nanoliposomal irinotecan was associated with better health-related QoL metrics [16].

Daraxonrasib represents a promising novel agent in the management of APC. In addition to achieving significant improvements in PFS and OS, it has also proven effective in pain control. In its pivotal registration trial including 500 patients, the median time to pain progression was 3.7 months in patients receiving control chemotherapy, compared with 9.2 months in those treated with daraxonrasib [17].

Collectively, these data demonstrate that widely used chemotherapeutic regimens are effective in achieving pain control across all stages of pancreatic cancer.

### 3.2. Radiation Therapy

Radiation therapy represents an effective, non-invasive treatment option for locally advanced or oligometastatic malignancies [18]. Although pancreatic radiotherapy remains technically challenging owing to the organ’s anatomical proximity to radiosensitive structures and physiological motion caused by respiration and peristalsis, recent technological advancements enable the delivery of higher, more targeted effective doses, ultimately enhancing its analgesic effect. Several studies have shown that targeted radiation therapy modalities effectively prolong OS and PFS in patients with locally advanced pancreatic cancer (LAPC) [19,20].

Some research groups have also included the alleviation of pancreatic cancer-associated pain as a study endpoint, which are described in detail below: A recent publication reported on patients who underwent daily MR-guided stereotactic body radiation therapy (SBRT) delivering 40–50 Gy in 5 fractions, prioritizing the sparing of adjacent gastrointestinal organs. A secondary endpoint of the study was pain control efficacy. At 3 months post-radiotherapy, 97% of patients experienced pain relief compared to baseline, and 82% reported an improved QoL [19] (Table 1).

Barry et al. evaluated 90 patients suffering from retroperitoneal tumor-related pain. Pancreatic cancer accounted for the majority of cases (90%), with a baseline mean daily intravenous morphine-equivalent consumption of 35.9 mg (SD: 67.6). Patients received single-fraction radiosurgery (25 Gy) targeting the coeliac plexus. The primary study endpoints included QoL improvement and the extent of pain reduction achieved with radiotherapy. Results showed that 53% of patients achieved at least a partial pain response at 3 weeks; opioid consumption remained stable at 3 weeks post-treatment and decreased by 30% at 6 weeks [21].

A multicenter, single-arm, phase 2 study was conducted across five countries. Ninety patients were enrolled, eighty-three of whom (92%) had pancreatic cancer; all received a single fraction of 25 Gy external-beam photons targeting the coeliac plexus. The primary endpoint was defined as a complete or partial pain response, specified as a ≥2 point reduction from baseline in the Brief Pain Inventory–Short Form (BPI-SF) average pain score at 3 weeks post-treatment. The investigators found that 53% of patients achieved at least a partial pain response, and these participants were also able to reduce their average daily opioid intake by 5.3 mg [22].

In a prospective, phase 2, single-center, nonrandomized trial, patients got a short-course radiation therapy of 24 Gy in 3 fractions of 8 Gy, once weekly (on day 1, 8, and 15). Before the treatment, all patients had a refractory pancreatic cancer-associated pain score of ≥5 (0–10 scale), which was difficult to manage with oral opioids or coeliac plexus block. Out of 29 patients, 24 (80.0%) reported a pain reduction of ≥2 points, and 16 (55.2%) were able to decrease their analgesic dose [23].

A Chinese study enrolled 22 patients with locoregional recurrent pancreatic cancer following surgery. Sixteen patients experienced pain prior to treatment, and a 100% pain response rate was achieved with SBRT [24].

Photon radiation and MR-guided SBRT are currently promising, investigational treatment modalities.

## 4. Palliative and Pharmacological Management

### 4.1. The Aim of Palliative Care

Pancreatic cancer and its treatments impose a high physical and psychosocial burden. Early palliative care may be implemented at the time of cancer diagnosis. This is particularly relevant in pancreatic cancer, where investigations are often initiated due to pain symptoms. Within the first eight weeks following cancer diagnosis, two-thirds of patients experience severe symptoms and a reduced quality of life [25]. Patients receiving early multidisciplinary palliative care according to their needs experience a significantly higher quality of life compared to patients on standard oncological treatment, as measured by the ESAS (Edmonton Symptom Assessment System) [26].

Palliative care provides continuous support for the patient and the caregivers (including family members and healthcare professionals) throughout the oncological and/or surgical treatment [27].

The palliative intervention naturally intensifies at the end of life, transitioning into hospice care. A large-scale study involving over 21,000 cancer patients evaluated pain during the final weeks of life, revealing that the highest prevalence of pain occurred in those with pancreatic cancer (86%). Structured pain assessment was significantly associated with higher odds of complete pain relief. Complete pain relief was significantly more likely in patients who died within a specialized palliative care setting or received specialized palliative home care, compared to those in standard care environments [28].

In the palliative concept, pain is tightly intertwined with the psychological, social, and spiritual state of the individual; hence, this framework known as “total pain” requires a multidisciplinary and comprehensive approach. A study involving 378 gastrointestinal cancer patients exhibited a significantly higher level of anxiety and a greater catastrophizing attitude, which impaired their coping mechanism for pain of those having pancreatic cancer [29].

### 4.2. Pharmacological Intervention

Pancreatic cancer-associated pain can be triggered by the tumor itself, by the tissues infiltrated from the growing tumor, and by the treatment (surgery and chemotherapy). Visceral and/or neuropathic pain in the upper abdominal region radiating to the back is typical, as well as abdominal discomfort due to decreased levels of digestive enzymes. In addition, pancreatic cancer-associated pain can be debilitating, making it challenging to manage the disease [30].

A comprehensive pain assessment including intensity and quality is essential before introducing any pain management strategy. Questionnaires, such as Numeric Rating Scale (NRS), Pain Management Index, European Organization for the Research and Treatment of Cancer Quality of Life Questionnaire, Barriers Questionnaire II, Medication Adherence Rating Scale, and Hospital Anxiety and Depression Scale, can be used. Clinical studies examining the potential use of AI in the pain assessment and management for patients living with colorectal cancer have promising results regarding pain reduction that may be implemented in pancreatic patient care [31].

Non-opioids (such as NSAIDs, metamizole, and paracetamol), opioids, and adjuvant analgesics (especially for neuropathic pain) such as antidepressants, anticonvulsants, and lidocaine are the cornerstone of cancer pain management. While the WHO analgesic ladder is the standard in pain management, combining it with the Plan-Do-Check-Act cycle (PDCA) method allows for more specific and individualized patient care [32].

Nonopioid analgesics are indicated for mild pain and are also effective as multi-modal adjuvants when combined with opioid therapy. Their usability is limited by a dose plateau effect and potential cardiovascular and gastrointestinal toxicities. Tramadol as a minor opioid is a weak mu-opioid receptor agonist with some norepinephrine and serotonin reuptake inhibition that is indicated for treating moderate pain. It should be used with caution in patients treated with other serotonergic or monoamine oxidase inhibitor-like medications (e.g., antidepressants) due to risk of serotonin syndrome.

For moderate-to-severe cancer pain, strong opioids are generally the mainstay of pharmacological treatment. Morphine, oxycodone, hydromorphone, and fentanyl are commonly used, while the choice of opioid and route of administration should be individualized according to clinical condition, organ function, previous opioid exposure, and the ability to use the oral route. Oral administration is generally preferred when feasible, whereas transdermal fentanyl may be useful in patients with stable opioid requirements who cannot reliably take oral medication. Morphine and oxycodone require particular caution in renal impairment because active metabolites or the parent drug may accumulate, whereas fentanyl is generally considered a more suitable option in significant renal dysfunction. Opioid doses should be titrated against analgesic response and adverse effects rather than to a predefined maximal dose. When adequate analgesia cannot be achieved because of dose-limiting toxicity, opioid rotation to an alternative opioid may improve the balance between analgesia and tolerability. Major opioids are used in severe pain; morphine and oxycodone should be used with caution in patients with decreased renal function due to the risk of metabolite accumulation. However, fentanyl seems safer in these cases. According to a study, opioid prescription rates for pancreatic cancer patients were higher among younger, female patients, those undergoing cancer-directed treatment and with longer expected survival times, whereas advanced age and comorbidities were associated with lower prescription rates. The utilization of palliative care was strongly associated with a higher frequency of major opioid prescriptions to manage severe, advanced-stage pain [33].

Breakthrough cancer pain should be specifically assessed because transient exacerbations may persist despite otherwise adequately controlled background pain. Management includes optimization of baseline analgesia and provision of an appropriate rescue opioid. In opioid-tolerant patients, immediate-release oral opioids can be used for predictable or longer-lasting episodes, whereas rapid-onset transmucosal fentanyl formulations may be considered for rapidly developing breakthrough pain because their pharmacokinetic profile more closely matches the short time course of many breakthrough episodes. Rescue treatment should be individually titrated, and frequent breakthrough episodes should prompt reassessment of the background analgesic regimen and of potentially treatable causes of pain.

Opioid-related adverse effects should be anticipated and actively managed, as they can substantially limit treatment adherence and quality of life. Constipation is particularly common and usually persists during long-term therapy; therefore, prophylactic laxative treatment should generally accompany regular opioid administration. Nausea and vomiting are common during initiation or dose escalation and may require temporary antiemetic therapy. Sedation, cognitive impairment, pruritus, urinary retention, and opioid-induced neurotoxicity may also occur. Persistent or severe toxicity should prompt evaluation of reversible contributing factors, dose adjustment, or opioid rotation. In selected patients with opioid-induced constipation that remains refractory to conventional laxatives, peripherally acting μ-opioid receptor antagonists may be considered.

Methadone is a μ-opioid receptor agonist with additional NMDA-receptor antagonistic and monoaminergic properties, which may make it particularly useful in selected patients with complex mixed nociceptive–neuropathic pain [34]. Studies show that even alongside ongoing opioid therapy, the addition of low-dose methadone (5 to 10 mg per day) is highly effective in managing complex (visceral and neuropathic) pain [35,36]. Adjuvant analgesics are particularly important when pancreatic cancer-associated pain has a neuropathic or mixed component. Gabapentinoids such as pregabalin and gabapentin and antidepressants including duloxetine may be considered for neuropathic pain, although treatment should be individualized because dizziness, somnolence, and other adverse effects may become clinically relevant in frail patients. Corticosteroids may provide short-term benefit in selected patients when pain is associated with tumor-related inflammation, edema, nerve compression, or capsular distension, although prolonged treatment is limited by systemic adverse effects. While the efficacy of anticonvulsants and antidepressants (such as pregabalin and duloxetine) in opioid-refractory neuropathic cancer pain management is well-established, an ongoing international, randomized trial is currently investigating these drugs in palliative care [37]. Intravenous lidocaine may relieve refractory neuropathic pain associated with pancreatic cancer. Recent pilot data in pancreatic ductal adenocarcinoma (PDAC) patients confirmed the treatment is feasible and safe, with only transient, minor side effects, while approximately one-third of patients achieved a significant analgesic response lasting up to one month [38].

Pancreatic exocrine insufficiency leads to severe maldigestion, which is a major driver of abdominal discomfort in over 80% of pancreatic cancer patients, even at the time of diagnosis. Implementing pancreatic enzyme replacement therapy (PERT) directly mitigates the maldigestion-induced pain and diarrhea, thereby significantly improving quality of life [39,40,41]. A large-scale study of 5887 patients demonstrated that integrating PERT into best practices significantly increased one-year survival rates from 34% to 45% [42].

### 4.3. Psychosocial Support in Total Pain

Pancreatic cancer is characterized by an exceptionally high symptom burden and rapid disease progression, leading to profound psychological, social, and spiritual distress that frequently exceeds levels observed in other malignancies, which significantly impairs both cognitive and emotional functioning, thereby compromising the patient’s effective coping strategies (Figure 1). Patients find the unpredictable and fluctuating nature of their symptoms more distressing than the symptoms themselves, as it severely limits social activities. Physical and psychological symptoms are deeply connected: fatigue triggers anxiety, and pain exacerbates hopelessness [43]. Total pain treatment requires a multidisciplinary approach including psychologists, social workers, dietitians, and physiotherapists alongside oncologists and palliative specialists; hence, ideally, specialized centers for pancreatic cancer patients should be created [44,45]. Depression affects 30–50% of pancreatic cancer patients often as a prodromal symptom. Since depression and cancer symptoms overlap (e.g., fatigue, weight loss), it frequently delays diagnosis and appropriate treatments, leading to lower survival rates. During an abdominal operation such as a pancreatectomy, patients with high psychosocial vulnerability experience worse post-surgery outcomes, including higher mortality and rehospitalization [46].

Spiritual well-being in cancer populations involves feeling that life has meaning, experiencing a sense of peace despite illness, and finding comfort in faith or beliefs. These values are part of the experience for both religious and non-religious patients, and are closely associated with the pain experience. Meaning-Centered Pain Coping Skills Training (MCPC) integrates two efficient psychosocial interventions: cognitive-behavioral therapy for pain management and meaning-centered therapy to increase spiritual well-being in the context of advanced cancer. The therapist seeks to improve patient’s self-efficacy for pain management through coping skills practices, guided imagery, activity-recovery cycles, brief relaxation practices, and values-based goal-setting techniques. In a recent study, MCPC participants reported better scores in pain severity, pain interference, and pain self-efficacy [47].

The need to provide psychosocial and palliative support for frail cancer patients at home led to significant improvements in telemedicine care for patients and their families. Studies show that telemedicine interventions are effective in managing distress and anxiety, while also addressing certain physical symptoms [48]. A European project examines the effect of a digitally assisted supportive care intervention on the quality of life of patients with pancreatic cancer, aimed at improving early access to multidisciplinary care. Although the intervention includes the guidance and monitoring of physical symptoms (e.g., pain) via smartwatches and mobile apps, further research is required on such interventions to address psychosocial support goals [49]. A QoL indicates that, while online self-management interventions for cancer survivors with chronic pain offer benefits like flexibility and validation of coping, engagement is often hindered by rigid scheduling, physical and cognitive limitations [50].

A Danish focus group study involving pancreatic cancer patients and their caregivers demonstrates that the patients’ unmet needs include psychological support and information beyond standard treatment protocols [51].

As cancer survival rates rise, more patients experience chronic pain. Chronic pain is also highly prevalent among cancer survivors, caused by treatments or pre-existing conditions. Long-term opioid therapy raises concerns due to its limited efficacy and high risk of side effects, tolerance, addiction, and overdose. Inadequate relief causes pain catastrophizing, which directly worsen the pain experience strongly associated with depression. Therefore, non-pharmacological therapies like cognitive behavioral therapy (CBT) can be used either alongside medication or as standalone treatment [52,53]. Music also has a pain-reducing effect in chronic cancer-related pain. This can be introduced alongside ongoing therapy, ranging from music listening initiated even by patients to music therapy offered by a therapist. A recent study shows that cancer patients prefer music therapy over listening to music due to the support experienced through joint music-making with the therapist, which correlates with lower pain intensity. Self-efficacy was found to be a significant mediator of music therapy for pain intensity and pain interference [54].

### 4.4. Palliative Sedation

Eighty percent of patients with advanced cancer experience severe pain at the end of life. Cancer patients in palliative care are adequately treated with moderate doses of opioids and only about 10% need daily morphine doses over 600 mg. At the end of life, continuous subcutaneous infusions (CSCIs) may be introduced to continue pain control. In a recent study involving pancreatic cancer, patients with uncontrolled pain CSCI were found to be effective in alleviating severe pain and anxiety [55].

Even with comprehensive palliative care, some patients with life-limiting diseases suffer from severe symptoms at the end of life, for which conventional treatments are not effective. In these cases, palliative sedation or intentional sedation may be indicated. The European Association for Palliative Care (EAPC) developed a framework (revised in 2023) on palliative sedation. The administration of sedative substances (primarily benzodiazepines) needs to be titrated to the lowest dosage that will provide adequate relief of suffering and maintain interactive cognitive function as far as possible. For patients with a better ECOG performance status, intermittent sedation may be more appropriate than continuous sedation to provide temporary relief whilst waiting for treatment benefit from other therapeutic approaches. Whenever possible, the physician needs to discuss with the patient the form of sedation that is appropriate to their individual status. Apart from emergency situations at the very end of life (such as massive bleeding, severe dyspnea, or refractory terminal delirium), a planned interruption of the sedation is recommended [56]. In an international multicenter study (PalSed), 78 adult patients with advanced cancer were monitored for discomfort (with the DS-DAT score) and depth of sedation levels (with the RASS-PAL score) during palliative sedation. Palliative sedation was initiated mostly for dyspnea, delirium, and exhaustion. The discomfort scores showed a significant decrease after the introduction of sedation. In this study, for most cases, palliative sedation was started directly as continuous sedation [57].

Continuous deep sedation (CDS) aims to decrease suffering by inducing unconsciousness until the patient’s natural death occurs. While CDS plays an appropriate role in end-of-life care, relatives often worry about the patient’s potentially remaining symptoms under sedation or whether CDS hastens death, which increases their own distress. The degree of relative’s involvement in decision-making may influence their emotional adjustment. When family members request the initiation of sedation, they may experience an intensified sense of moral responsibility and distress. A French multicenter study (part of the APSY-SED research project) investigated the psychological adjustment over time among relatives and healthcare professionals caring for cancer patients for whom CDS is considered. It followed a longitudinal protocol with data collection at three time points: at the patient’s death, at three months post-death, and at six months post-death, and only for relatives, thirteen months post-death. Results showed that relatives were more satisfied with the care and showed lower depressive symptoms if CDS were patient-indicated. Healthcare professionals showed higher job satisfaction and experienced little job strain when CDS was started due to the feeling of effectively alleviate patient suffering [58]. To prevent sedation-related distress among relatives and caregivers, clear and educational communication is essential with relatives and healthcare team members about the process and their concerns.

## 5. Interventional Pain Management

### 5.1. Coeliac Plexus Neurolysis

Coeliac plexus neurolysis (CPN) is an interventional procedure where a neurolytic agent (most commonly alcohol) is injected in the vicinity of the coeliac plexus, thus damaging the fibers responsible for the visceral pain signals triggered by the tumor (Figure 2). This technique is known to alleviate not only upper abdominal pain but back pain [59]. Major cancer pain guidelines recommend CPN for pancreatic cancer cases when optimized pharmacological regimens fail to provide adequate analgesia and/or intolerable side effects are present [8,34]. CPN can be performed using percutaneous image-guided, endoscopic ultrasound (EUS)-guided, or intraoperative approaches, which differ primarily in access route, clinical setting, and patient selection. Percutaneous CPN, generally performed under fluoroscopic or CT guidance, is widely applicable and may be particularly suitable for patients referred directly for interventional pain management, whereas EUS-guided CPN provides direct visualization of the coeliac region from the stomach and can conveniently be performed during diagnostic or therapeutic endoscopy. Intraoperative CPN may be considered when the abdominal cavity is already accessed surgically, but its role has diminished with the availability of less invasive techniques. CPN offers superior analgesia when compared with pharmacological pain management alone; however QoL and OS rates are similar in both groups [60]. A recent systematic review by Okita et al. tried to compare the different techniques for CPN (such as endoscopic-guided, intraoperative, and percutaneous techniques) and they concluded that addition of CPN significantly improved pain intensity scores at 4 weeks, with non-significant improvement at 8 and 12 weeks, although the improvement was significant at 4 and 8 weeks in a supplementary analysis, but comparison of each individual group was difficult due to the high risk of bias for the majority of publications [61]. Current evidence does not demonstrate clear superiority of one approach: A randomized trial by Yoon et al. compared endoscopic ultrasound guided versus percutaneous CPN for pancreatic cancer patients in a randomized clinical trial and found that both procedures were safe and provided sustained decrease in pain scores up to 3 months postintervention. Based on their data, they recommend both procedures depending on the resources and expertise of other centers [59]. CPN should therefore be considered when pancreatic cancer-associated visceral pain remains inadequately controlled or pharmacological treatment causes unacceptable adverse effects, with the choice and timing of technique guided by anatomy, clinical setting, available expertise, and expected prognosis. Across approaches, potential adverse events include transient hypotension, diarrhea, and procedure-related pain, while serious neurological, vascular, or infectious complications are rare; incomplete or temporary analgesia and limited efficacy for predominantly somatic or neuropathic pain remain important limitations.

### 5.2. Splanchnic Nerve Neurolysis

Splanchnic nerve neurolysis (SNN) is an invasive procedure where the pain pathway is disrupted proximal to the coeliac plexus. There are three major splanchnic nerves in the body: major splanchnic nerve, minor splanchnic nerve, and lesser splanchnic nerve arising from the T5–9, T10–11, and T12 spinal nerves, respectively. SNN may be carried out via a thoracoscopic approach and as a fluoroscopic- or CT-guided percutaneous procedure at the T10–T12 vertebral body using radiofrequency ablation (RFA) or alcohol neurolysis [62,63,64]. Several clinical trials examined the efficacy of SNN in pancreatic cancer patients and compared the mode of ablation. In a prospective trial, Grigoriadis et al. found that the mean pain score prior to RFA neurolysis of the splanchnic nerves was severe (9.0 NVS, numeric verbal scale units), which was reduced significantly to mild and moderate pain, measured by NVS units, in both the short and long term, up to 1 year. SNN also significantly reduced analgesic need in most patients [63]. In a randomized controlled trial, Amr et al. found that SNN performed by RFA is more effective and gives longer lasting results with lower opioid consumption when compared to alcohol neurolysis [63]. In a recent meta-analysis, Lu et al. compared CPN to SNN and found no significant difference in pain scores after 2 weeks to 6 months between the two procedures; however, in the SNN group, opioid consumption was lower with fewer side effects. The same article highlights that SNN may be carried out even in disturbed anatomical settings, where CPN might be difficult or even impossible to complete. In addition, they emphasized the importance of early supportive care in order to improve mood and QOL in patients with advanced cancer [65]. Importantly, SNN may be carried out as a salvage approach when CPN fails to provide proper pain relief [66].

### 5.3. Spinal Opioids

Spinal opioids may be administered by epidural (ED) or intrathecal (IT) routes either with percutaneous or tunneled catheters and by external or by implantable pumps. Opioids administered spinally readily bind to the mu receptors of substantia gelatinosa, thus potentially reducing opioid consumption. Opioids administered by the IT route has the highest efficacy; 1 mg of IT morphine equals 24 mg of ED and 300 mg oral morphine. Reduced opioid doses used spinally lead to fewer systemic side effects. Adjuvant drugs such as local anesthetics, clonidine, and ziconotide are used frequently to enhance the effect of the opioid medications [8]. Major cancer pain guidelines recommend spinal opioid therapies for patients experiencing inadequate pain control, intolerable sedation, confusion, and constipation from systemic analgesics [8,34]. The European Society of Medical Oncology recommends using an implantable device in cases when life expectancy of the patient exceeds 6 months, given the higher risk of implantation procedure and the high cost of these devices. The guideline also recommends spinal therapies to be performed and monitored by a skilled team as part of the cancer pain management strategy, which also highlights the importance of multidisciplinary teamwork, as well as the need for early collaborative supportive care [8]. In an observational follow-up study of IT drug delivery systems in patients with pancreatic cancer, implantable pumps and subcutaneous port delivery systems were compared. The study found significantly reduced pain scores at 1 week and 1 and 3 months as well. Authors concluded that long-term IT drug delivery is both highly efficacious and safe [67]. In a retrospective observational study including patients with pancreatic cancer, it has been concluded that ED analgesia is an effective pain control strategy in terminally ill cancer patients, which is able to significantly reduce the need for opioids in this population [68].

### 5.4. Pain Management in Metastatic Disease

A retrospective analysis by Puri et al. found that the most common sites of metastases in pancreatic adenocarcinoma are the thoracic and lumbar vertebrae, where the majority of these lesions are osteolytic [69]. Vertebral augmentation procedures such as vertebroplasty or kyphoplasty are known for relieving mechanical back pain caused by vertebrae compression fractures. A systematic review on vertebral augmentation in cancer metastases found that the mean pain intensity scores significantly reduced within 48 h with a similar trend towards decreased analgesic use [70]. Percutaneous chordotomy is a minimally invasive, CT-guided procedure where the lateral spinothalamic tract is ablated with radiofrequency, thus relieving contralateral pain. Chordotomy may be used in cases of unilateral pain triggered by skeletal metastases or in cases of plexus compressions. A retrospective analysis of 207 chordotomy patients found a 92.5% initial success rate in the malignancy group in terms of pain scores [71]. In a case series of 15 individuals suffering from vertebral metastatic pain, a dorsal root pulsed radiofrequency procedure provided sound pain relief [72].

## 6. Practical Advices for an Integrated Care Pathway for Pancreatic Cancer-Associated Pain

An essential prerequisite for effective pancreatic cancer-associated pain management is the integration of oncology, palliative care, and interventional pain medicine into a coordinated multidisciplinary team (Figure 3). Regular multidisciplinary discussions can facilitate patient selection, clarify the contribution of each specialty and identify gaps or delays within the patient pathway.

Routine screening for pain and related symptoms using validated questionnaires may be incorporated into regular oncology follow-up. Palliative care specialists can play a central role in optimizing pharmacological analgesia, opioid rotation, adjuvant therapy, and psychosocial support. Patients with severe or refractory pain should be referred early to physicians experienced in interventional cancer pain management. These roles should remain flexible and overlapping within an integrated, patient-centered model.

Implementation must account for local regulations, reimbursement, medication availability, institutional expertise, and access to interventions. Intrathecal drug delivery systems, for example, may not be reimbursed or readily available in many healthcare systems. In such settings, teams should adapt treatment pathways to available alternatives, including long-term epidural analgesia, endoscopic coeliac plexus interventions, or thoracoscopic splanchnicectomy. Similarly, differences in opioid availability may restrict opioid rotation, emphasizing the importance of adjuvant analgesics and interventional strategies.

A mechanism-based pain assessment should precede treatment selection, as pancreatic cancer-associated pain may present with visceral, somatic, neuropathic, or mixed features and may change throughout the disease course. Visceral pain is typically related to the primary tumor and may favor coeliac plexus or splanchnic nerve interventions, whereas somatic pain from local invasion or metastases requires treatment directed at the affected structure. Neuropathic pain caused by neural invasion, radiculopathy, or treatment-related nerve injury may require adjuvant pharmacotherapy and selected neural interventions. Treatment-related pain should be distinguished from tumor progression, while breakthrough pain requires optimization of baseline analgesia together with appropriate rescue treatment. In advanced and end-of-life disease, assessment should additionally consider prognosis, functional status, symptom burden, and the time required to benefit from an intervention. As these mechanisms frequently coexist, repeated pain reassessment is essential to guide escalation from pharmacological treatment to mechanism-specific interventional strategies.

Each patient should be treated individually, but there can be an optimal strategy for every stage of the disease.

Pain management should begin early and should not necessarily await histological confirmation or completion of staging and oncological treatment planning. Pharmacological analgesia should be initiated in parallel with the diagnostic process, while potentially treatable structural causes, such as pancreatic or biliary duct obstruction, should be identified and addressed. When pain remains difficult to control, early interventional assessment may be appropriate. Thus, diagnostic evaluation, oncological treatment, palliative care, and pain management should ideally proceed in parallel rather than as strictly sequential steps.

Patients with confirmed pancreatic cancer who are not candidates for surgical resection generally receive systemic anticancer therapy, which may itself reduce pain through tumor control. If pain persists, pharmacological therapy should be initiated or optimized, with early palliative care involvement in difficult-to-control cases. CPN may be considered for predominantly visceral upper abdominal pain, while SNN may represent an alternative in patients with inadequate pain relief following CPN.

Following pancreatic surgery, new or worsening pain associated with local recurrence may have both visceral and somatic components. Postoperative anatomical alterations may also affect the feasibility and efficacy of CPN; therefore, splanchnic nerve interventions may be considered as an alternative. Severe refractory pain may require neuraxial strategies, including epidural or intrathecal analgesia. Close palliative care involvement is particularly valuable when combinations of opioids, non-opioid analgesics, and adjuvant agents are required.

In advanced or metastatic disease, palliative care is essential for both symptom management and psychosocial support for patients and their families. Refractory pain should prompt consideration of advanced interventional strategies rather than repeated escalation of systemic analgesics alone. Depending on the pain mechanism, disease distribution, prognosis, and available expertise, options may include neuraxial or intrathecal analgesia and targeted procedures such as vertebral augmentation for painful vertebral metastases or percutaneous cordotomy in carefully selected patients with severe, predominantly unilateral refractory cancer pain.

## 7. Conclusions

Pain is one of the most burdensome symptoms of pancreatic cancer which may arise from the primary tumor, disease progression, metastatic involvement, or anticancer treatments. Pain management therefore should rely on a multidisciplinary approach, where oncological care, palliative care, and pain medicine should all be integrated. Effective anticancer treatments like chemo- and radiotherapy can significantly reduce pain besides disease control, and therefore, it is an important component of pain management. Palliative care in terms of psychosocial support and optimized pharmacological therapy should be integrated throughout the disease trajectory. Coeliac plexus neurolysis and splanchnic nerve neurolysis can provide effective visceral pain relief, while spinal drug delivery and other targeted interventions may offer valuable options for refractory, treatment-related, or metastatic pain. Importantly, these therapeutic modalities should not be considered as consecutive steps of a rigid analgesic ladder; rather, they should be allocated to the patients needs. Ultimately, optimal pancreatic cancer-associated pain management requires continuous collaboration between oncology, palliative care, and pain specialists. A patient-centered pathway that dynamically integrates disease-directed, pharmacological, supportive, and interventional strategies throughout the disease course offers the most rational approach to reducing pain, limiting treatment burden, and preserving quality of life.

## Figures and Tables

**Figure 1:**
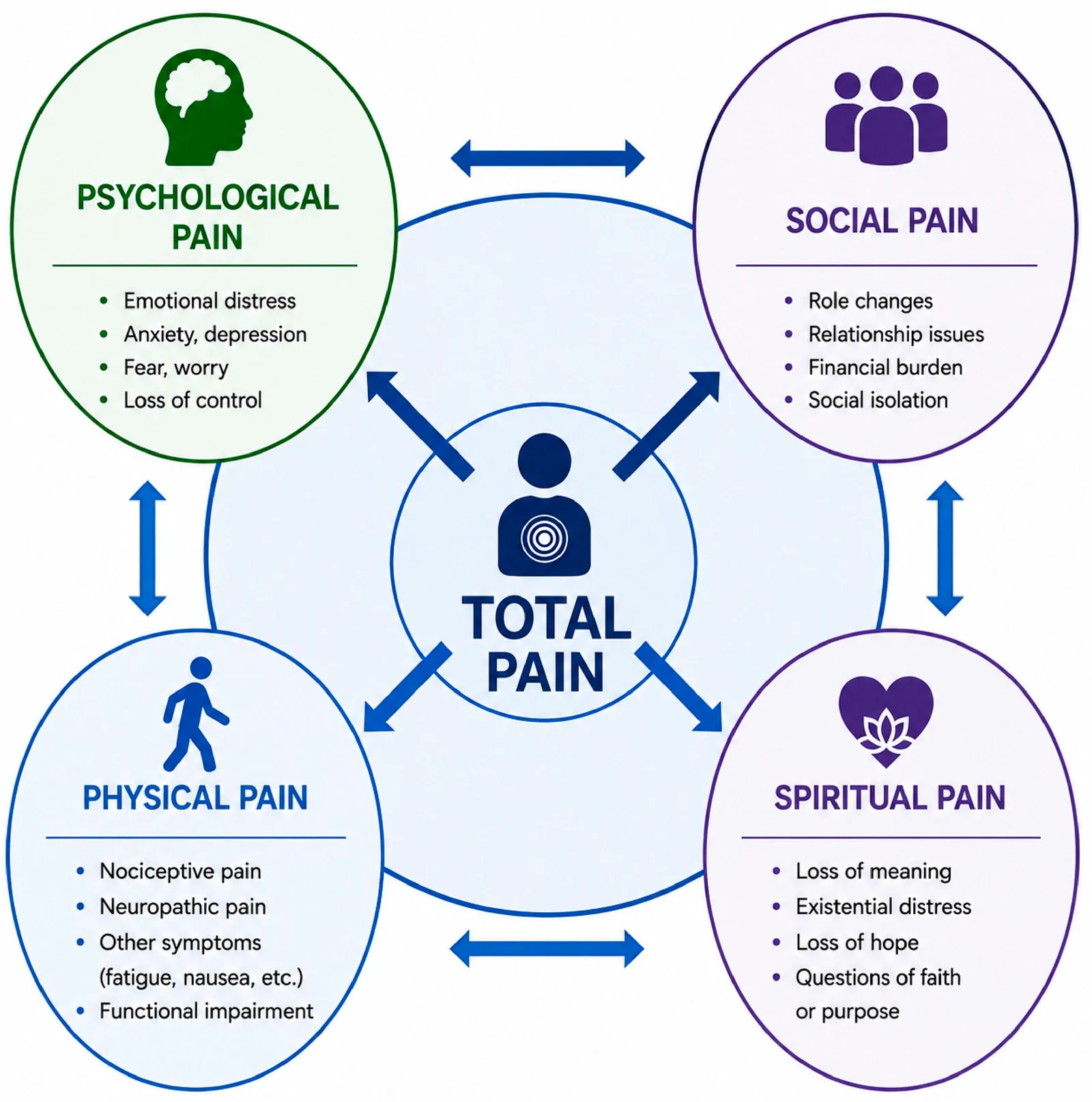
The concept of “total pain”.

**Figure 2:**
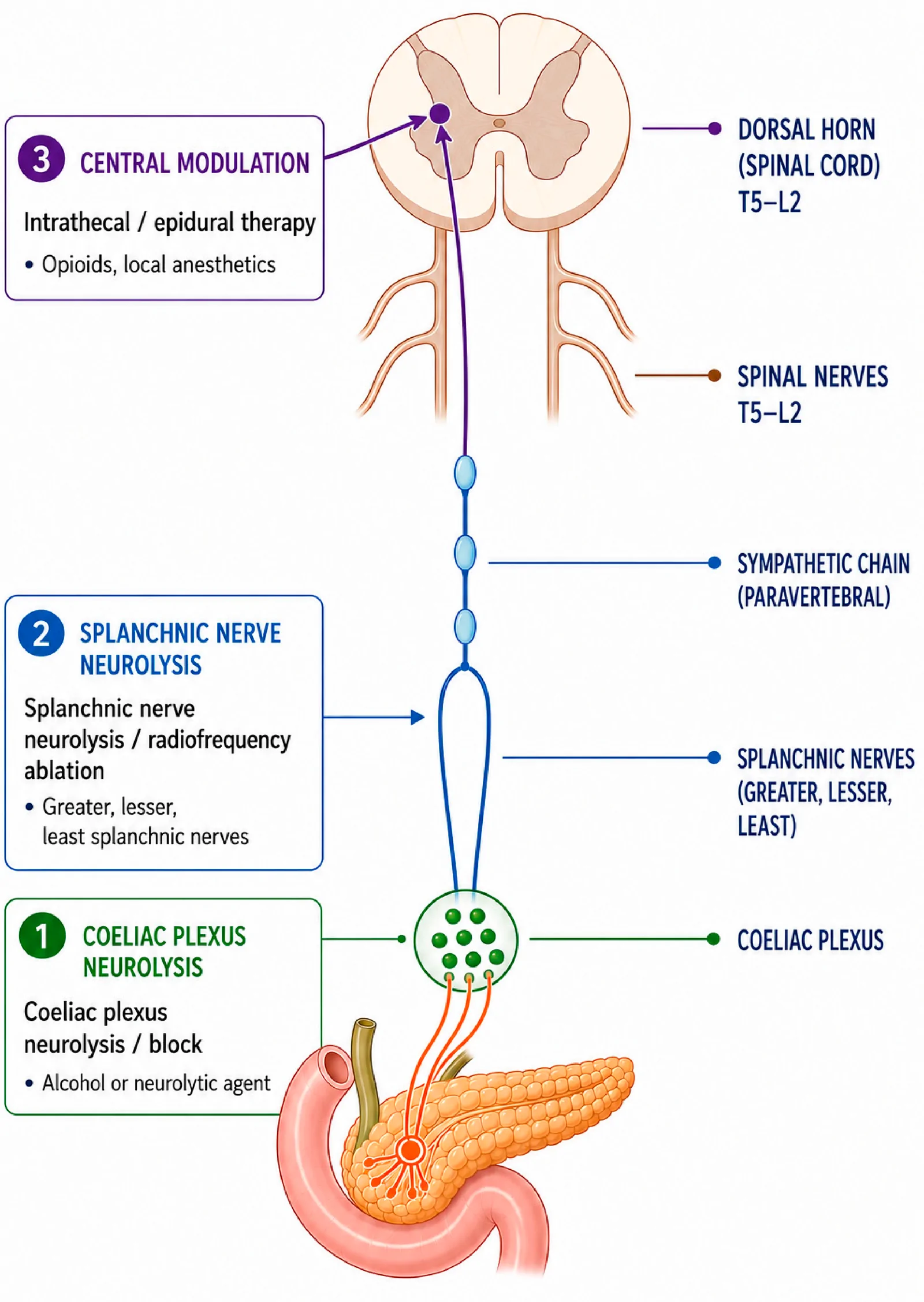
Visceral pain pathway and interventional targets.

**Figure 3:**
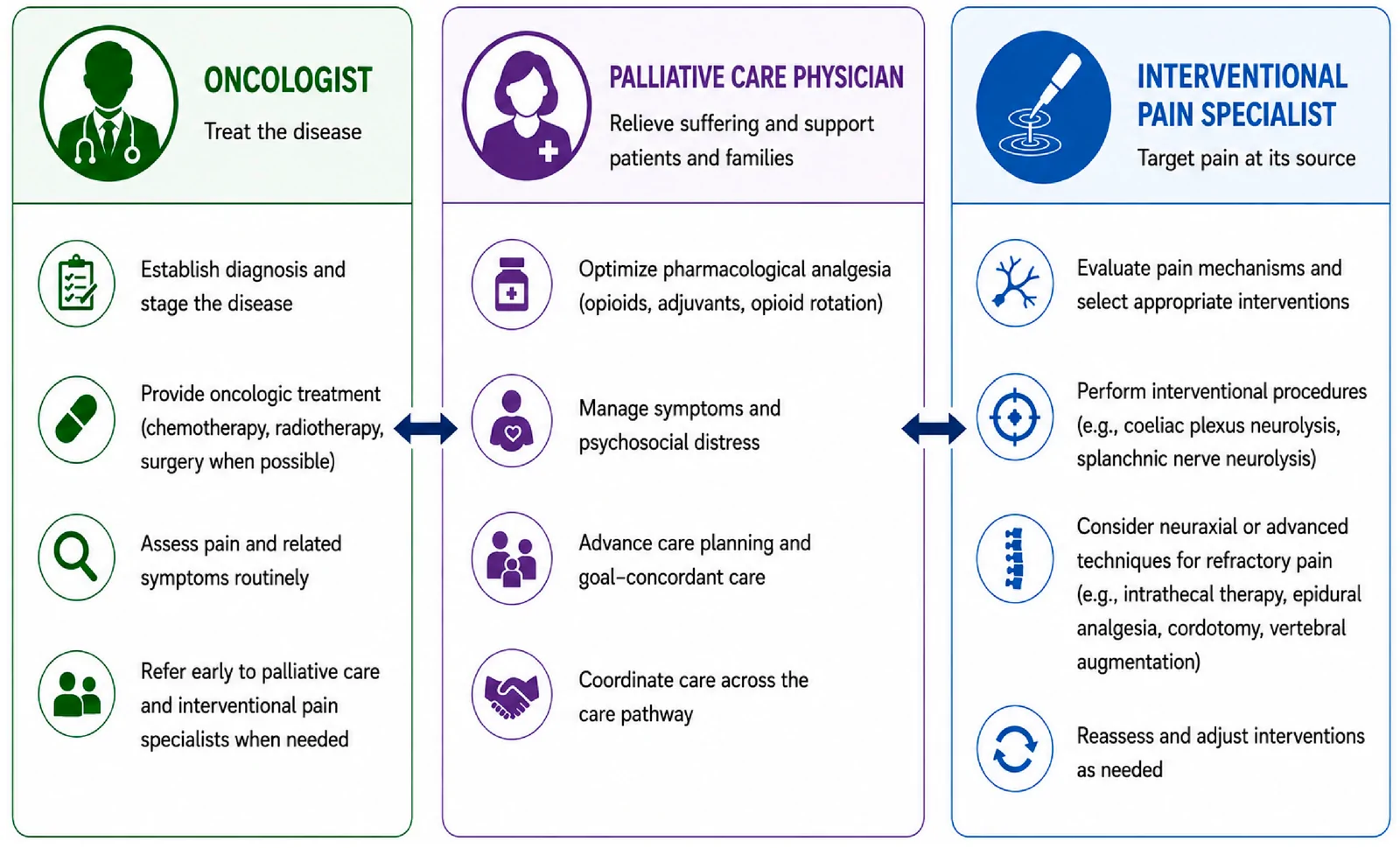
Collaborative approach to cancer pain management.

**Table 1:** Radiation therapy for pain relief.

Study/Reference	Radiation Type	Dose and Fractionation	Design, Patients No	Pain Endpoint	Pain Response
Wang, C et al. [19]	MR-guided stereotactic body radiation therapy (SBRT)	40–50 Gy in 5 fractions	Single-center, open-label study; 34 patients	NRS score	97% ≥2-point reduction
Barry et al. [21]	Single-fraction radiosurgery	25 Gy in 1 fraction	Multicenter, single-arm study; 90 patients	FACT-Hep scores	57% SS and CS improvement
Lawrence, Y.R. et al. [22]	External-beam photons	25 Gy in 1 fraction	Multicenter, single-arm, phase 2 study; 90 patients	BPI-SF average pain score	53% ≥2-point reduction
Tello Valverde, C.P. et al. [23]	Short-course radiation therapy	24 Gy in 3 fractions of 8 Gy (once weekly)	Single-center, phase 2, single-arm study; 30 patients	BPI score	55.2% ≥2-point reduction
Ji, X. et al. [24]	Stereotactic body radiation therapy (SBRT)	median dose of 40 Gy (range of 30–50 Gy)/4 to 7 fractions.	Retrospective, single-center study; 22 patients	VAS score	100% CS improvement

NRS—Numeric Rating Scale; SBRT—stereotactic body radiation therapy; FACT-Hep—Functional Assessment of Cancer Therapy—Hepatobiliary; BPI-SF—Brief Pain Inventory–Short Form; SS—statistically significant; CS—clinically significant; VAS—Visual Analogue Scale; EORTC—European Organization for Research and Treatment of Cancer; QLQ-C30—Core Quality of Life questionnaire C30; AI—artificial intelligence; MR—magnetic resonance; NMDA—N-methyl-D-aspartate receptor; DS-DAT—Discomfort Scale-Dementia of Alzheimer Type; RASS-PAL—Richmond Agitation-Sedation Scale—Palliative Version; CR—complete response; CT—computed tomography; NSAID—nonsteroidal anti-inflammatory drug.

## Data Availability

No new data were created or analyzed in this study. Data sharing is not applicable to this article. The literature sources are cited in the manuscript.

## Abbreviations

ECIS	European Cancer Information System
OS	overall survival
PFS	progression-free survival
NSCLC	non-small cell lung cancer
APC	advanced pancreatic cancer
CR	complete response
PR	partial response
PS	performance status
QoL	quality of life
VAS	Visual Analog Scale
LAPC	locally advanced pancreatic cancer
SBRT	stereotactic body radiation therapy
MR	magnetic resonance
CT	computed tomography
BPI-SF	Brief Pain Inventory–Short Form
ESAS	Edmonton Symptom Assessment System
NRS	Numeric Rating Scale
NSAID	non-steroidal anti-inflammatory drug
WHO	World Health Organization
PDCA	Plan-Do-Check-Act cycle
NMDA receptor	N-methyl-D-aspartate receptor
PDAC	pancreatic ductal adenocarcinoma
PERT	pancreatic enzyme replacement therapy
MCPC	Meaning-Centered Pain Coping Skills Training
CBT	cognitive behavioral therapy
CSCI	continuous subcutaneous infusions
EAPC	European Association for Palliative Care
CDS	continuous deep sedation
CPN	coeliac plexus neurolysis
SNN	splanchnic nerve neurolysis
RFA	radiofrequency ablation
NVS	numeric verbal scale
ED	epidural
IT	intrathecal
